# Takotsubo Cardiomyopathy Induced by Stress From Amyotrophic Lateral Sclerosis and a Mechanical Fall

**DOI:** 10.7759/cureus.47068

**Published:** 2023-10-15

**Authors:** Nicholas R Munoz, Chibuike C Agwuegbo, Ali Ghorbani, John M Vincent Coralde, Robin Abdelmalik

**Affiliations:** 1 Internal Medicine, Southwest Healthcare, Temecula, USA; 2 Internal Medicine, Southern California Medical Education Consortium, Temecula, USA; 3 Cardiology, Southwest Healthcare, Temecula, USA

**Keywords:** amyotrophic lateral sclerosis, takotsubo cardiomyopathy, coronary artery, mid ventricular ballooning syndrome, goal directed medical therapy, coronary angiography, takotsubo cardiomyopathy and ventricular arrhythmias, heart failure, cardiology research, cardiology

## Abstract

Named after the Japanese octopus trap, Takotsubo cardiomyopathy is an acute myocardial condition characterized by a reversible ventricular dysfunction with ballooning of the left ventricle (LV) during systole. A catecholamine surge is likely the primary mechanism responsible for myocardial damage in this condition. The association between amyotrophic lateral sclerosis (ALS) and Takotsubo cardiomyopathy has not been well established. We present a unique case of Takotsubo cardiomyopathy diagnosed in a patient with ALS who presented after a fall with shortness of breath, generalized weakness, and hypotension. She was found to have troponinemia, elevated brain natriuretic peptide, and Osborn waves without ST-segment changes noted on electrocardiography (EKG). The diagnosis of Takotsubo cardiomyopathy was confirmed via transthoracic echocardiography (TTE), which revealed reduced left ventricular ejection fraction, apical ballooning of the LV, akinesis of the ventricular apex, and hyperkinesis of the base of the heart. Coronary angiography revealed no coronary artery disease. She was managed medically and was hemodynamically stable at the time of discharge.

## Introduction

Takotsubo cardiomyopathy is a reversible, acute, structural myocardial condition distinguished by transient ventricular dysfunction that is not caused by obstructive coronary artery disease. First reported in Japan in 1990 by Sato et al., the condition derives its name from the unique ballooning shape of the left ventricle (LV) during systole, resembling a Japanese octopus' trap called "takotsubo". The pathophysiology of Takotsubo cardiomyopathy remains incompletely understood, but it is often triggered by acute severe emotional or physical stressors and primarily affects post-menopausal women, although cases have been reported in men and younger individuals as well [1]. 

While originally believed to be a benign condition with full recovery, some research has highlighted the potential complications of the condition, including ventricular arrhythmias, cardiogenic shock, and even death [2]. This case report presents a comprehensive analysis of a case of Takotsubo cardiomyopathy in a 69-year-old female with amyotrophic lateral sclerosis (ALS), providing a valuable understanding of its clinical presentation, diagnostic challenges, management strategies, and associated outcomes.

## Case presentation

**Figure 1:**
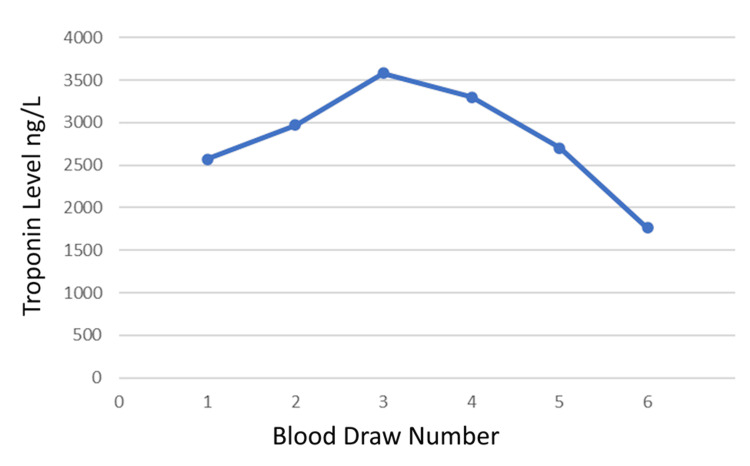
The patient's troponin levels during the first two days of hospitalization This figure indicates the patient's troponin levels rose, peaked, and fell indicating an acute myocardial injury. The troponin levels were drawn between two and five hours apart.

**Figure 2:**
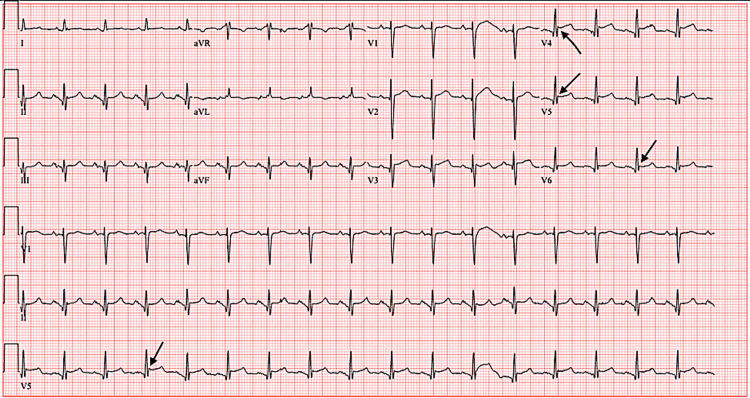
The patients' EKG showing Osborn waves in leads V4, V5, and V6 as indicated by arrows

The patient was a 69-year-old female with a past medical history of ALS (diagnosed three months previously), epilepsy, chronic obstructive pulmonary disease on home oxygen, and dysphagia who was admitted to the hospital after a mechanical fall complicated by hypotension. The patient was hospitalized two days prior for dysphagia with the placement of a percutaneous endoscopic gastrostomy (PEG) tube. Once home, she tripped over her PEG tube while ambulating to the bathroom. The patient landed on her buttocks and was on the floor for 45 minutes, as she was too weak to stand. Her doorbell rang and she crawled to the front door where her neighbor called emergency medical services. During this time, she felt extremely short of breath. The patient otherwise denied chest pain, diaphoresis, palpitations, fever, chills, headache, diarrhea, flu-like symptoms, abdominal pain, nausea, or vomiting. The patient reported that she was depressed and anxious. She stated she was dying from ALS and endorsed a feeling of hopelessness about her worsening symptoms of weakness and increasing shortness of breath. The patient was hypotensive with a blood pressure of 88/50, a heart rate of 110 bpm, and an oxygen saturation (SpO2) of 99% on 2 liters of nasal cannula oxygen. On physical exam, the patient had dry mucous membranes, sinus tachycardia, no murmurs, and clear lung sounds bilaterally. She was found to have a leukocyte count of 12,400 cells/mcL, a brain natriuretic peptide level of 247 pg/mL (peaking at 8,024 pg/mL), and an elevated troponin level of 2,570 ng/L (peaking at 3,303 ng/L) (Figure 1). Computed tomography angiography of the chest was performed showing moderate emphysema, bibasilar atelectasis or infiltrates, and no evidence of pulmonary embolism. EKG showed Osborn waves in the precordial leads, and sinus tachycardia without ST elevations (Figure 2). The patient remained hypotensive despite fluid boluses and was upgraded to the intensive care unit (ICU).

In the ICU, the patient was started on a heparin drip, vancomycin, and piperacillin-tazobactam. Her hypotension did not respond to an initial fluid bolus, so she was started on a norepinephrine drip. After aggressive intravenous hydration, her tachycardia and hypotension resolved, and the vasopressor was discontinued, as she no longer required norepinephrine to maintain a mean arterial pressure of greater than 65 mmHg. She received a blood culture, urine culture, and sputum culture that did not grow any bacteria. The patient’s leukocytosis resolved on the second day of hospitalization. Throughout the rest of her hospital stay, she had a normal heart rate and blood pressure. TTE was performed showing an LV ejection fraction of 40-45% with apical ballooning of the LV, akinesis of the ventricular apex, and hyperkinesis of the base of the heart (Video 1). The cardiology team was consulted due to the patient's new onset heart failure, and non-ST elevated myocardial infarction with a GRACE (the Global Registry of Acute Coronary Events) ACS (acute coronary syndrome) score of 164 points, indicating a 30% probability of death from admission to six months. The patient received a right and left coronary artery angiogram, which showed no evidence of coronary artery stenosis. She started aspirin 81 mg daily and atorvastatin 40 mg daily. Cardiology recommended starting goal-directed heart failure therapy with medication such as beta-blockers and angiotensin receptor inhibitors outpatient, as the patient was recently hypotensive. Goals of care were discussed, and the patient declined hospice. She stated she lived alone, had no nearby family, and no financial support for caretakers. The patient was medically stable, downgraded from the ICU, and discharged to a skilled nursing facility the following day.

**Video 1:** Echocardiogram of the patient's heart showing apical ballooning of the LV, akinesis of the ventricular apex, and hyperkinesis of the base of the heart LV: left ventricle

## Discussion

**Figure 3:**
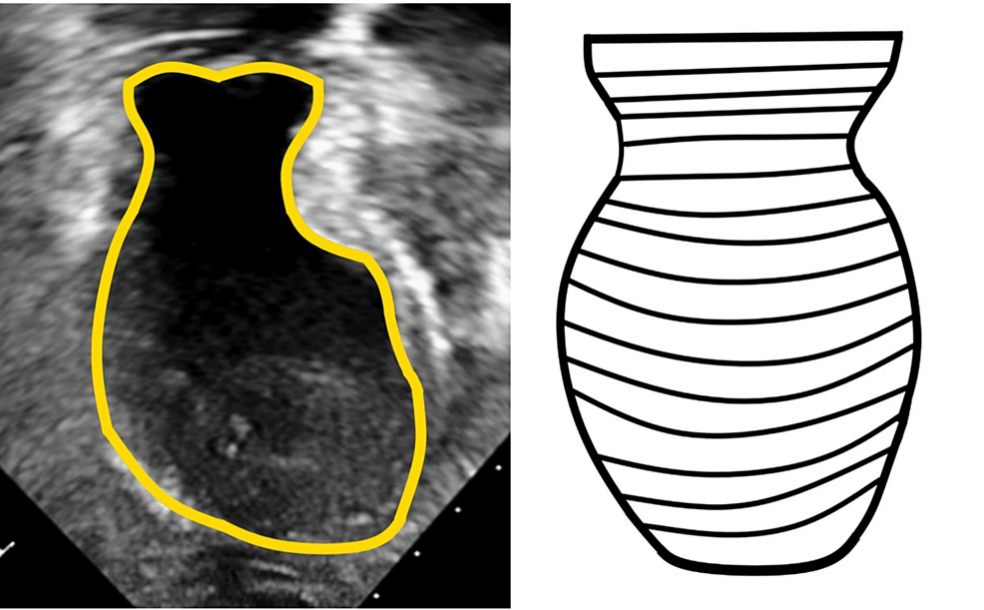
The patient's dilated LV is outlined in yellow (left), compared to an illustration of a Japanese Takotsubo octopus trap (right) Image Credits: Nicholas R. Munoz

Takotsubo cardiomyopathy is also known as Takotsubo syndrome, stress-induced cardiomyopathy, and apical ballooning syndrome. It is a reversible apical ballooning of the myocardium resembling a myocardial infarction that is not caused by coronary artery disease. The syndrome's name comes from a container used in Japan to catch octopus. The container has a ballooned bottom and a narrow neck resembling the heart of a patient with Takotsubo cardiomyopathy (Figure 3) [3]. About 90% of patients are women. Postulated mechanisms of the disease include coronary artery spasm, myocarditis, adrenergic storm, and dynamic mid-cavity cardiac obstruction [4].

The Mayo criteria is used to diagnose Takotsubo cardiomyopathy. This criterion includes temporary LV dysfunction during systole, absence of coronary artery disease (unless the wall motion abnormalities are not in the distribution of the diseased coronary artery), elevation of cardiac troponin or new onset EKG abnormalities, and absence of pheochromocytoma or myocarditis (Table 1). Diagnosis is often made after heart angiography shows patent coronary arteries [5]. The diagnostic study of choice to assess LV dysfunction is TTE. Imaging often shows ballooning of the myocardial apex with akinesia or dyskinesia of the apical and mid portion of the ventricle. Often, hyperkinesis at the base of the heart is seen [6]. EKG abnormalities can include Osborn waves, also known as J waves, which is a deflection at the R-ST junction thought to be a result of early repolarization [7].

**Table 1:** Mayo criteria for diagnosis of Takotsubo cardiomyopathy

Mayo Criteria for Diagnosis of Takotsubo Cardiomyopathy
Temporary LV systolic dysfunction.
Absence of CAD (unless the wall motion abnormalities are not in the distribution of the diseased coronary artery).
Elevation of cardiac troponin or new EKG abnormalities.
Absence of pheochromocytoma or myocarditis.

**Figure 4:**
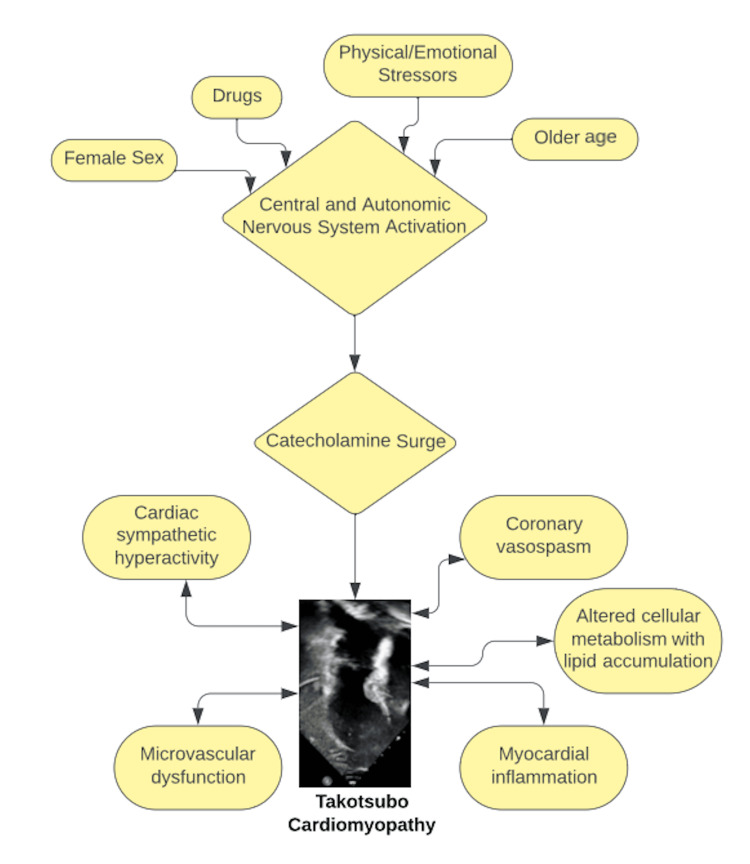
Pathophysiology of Takotsubo cardiomyopathy Image Credits: Chibuike C. Agwuegbo

Despite extensive research, the cause and pathogenesis of Takotsubo cardiomyopathy remain incompletely understood. Evidence indicates the etiology of the disease starts with acute stressors that induce thalamus activation, increasing cortisol and catecholamine levels in the bloodstream. Both circulating epinephrine and norepinephrine are significantly increased in the acute phase of Takotsubo cardiomyopathy. This catecholamine surge leads to catecholamine toxicity, adrenoceptor-mediated damage, epicardial and microvascular coronary artery vasoconstriction with or without spasm, and increased cardiac workload (Figure 4). Since the disease is most common in postmenopausal women, estrogen deprivation may play a facilitating role, likely mediated by endothelial dysfunction [6].

Although central nervous system disorders, such as stroke and epilepsy, are known to trigger Takotsubo cardiomyopathy, their relation to neurodegenerative disorders, such as ALS, is poorly understood. In a retrospective study of 250 patients with ALS and 870 patients with synucleinopathies (such as Parkinson's disease, dementia with Lewy bodies, and multiple system atrophy), four cases of Takotsubo cardiomyopathy were found in patients with ALS (1.6%), and none in the synucleinopathies group. Further literature review identified 16 Takotsubo cardiomyopathy cases associated with ALS. In these cases, 55% were female, 35% had a bulbar onset, and 45% had a limb onset. The mean age of Takotsubo cardiomyopathy onset was 63.3 ± 9.0 years, and the mean interval time from ALS onset to Takotsubo cardiomyopathy development was 4.9 ± 3.0 years [6].

According to the International Takotsubo Registry study of 1750 patients, the most common symptoms of Takotsubo cardiomyopathy are chest pain in 75.9% of patients, dyspnea in 46.9% of patients, and syncope in 7.7% of patients [6,8]. Patients can also develop heart failure and arrhythmias, including ventricular fibrillation, torsades de pointes, atrioventricular blocks, and bradycardia. Additionally, cardiogenic shock and cardiac arrest have been reported [6,9,10]. Congestive heart failure should be managed with goal-directed medical therapy, including beta-blockers, angiotensin receptor-neprilysin inhibitors, angiotensin receptor blockers, angiotensin-converting enzyme inhibitors, and aldosterone antagonists. Cardiogenic shock is managed with inotropes, vasopressors, and mechanical left ventricular assist devices (LVAD) [11]. Prophylactic anticoagulation helps prevent the development of left ventricular thrombi, which may embolize distally to organs. Arrhythmias may be managed with intravenous magnesium, temporary ventricular pacing, and elimination of QT-interval prolonging medications. Patients with Takotsubo cardiomyopathy typically have excellent recovery rates, up to 96%. In-hospital mortality rates ranged from 0-8%, and recurrence rates ranged from 0-15%. Risk factors associated with Takotsubo cardiomyopathy include age greater than 55 years old, female sex, left ventricular ejection fraction below 35%, and presence of arrhythmias. Close follow-up is recommended after hospital discharge, and patients should be followed until a demonstrable improvement in ventricular function is noted [12].

Physical triggers of Takotsubo cardiomyopathy are more common than emotional triggers, at 36.0% versus 27.7% [8]. It’s likely the emotional stress from the patient's rapidly progressing ALS, mechanical fall, and hospitalization caused the release of stress hormones leading to Takotsubo cardiomyopathy. The rise and fall of troponins during her hospitalization points to an acute etiology. The patient's tachycardia was likely secondary to dehydration as it resolved after adequate fluid resuscitation and did not recur. The patient did not have hypertension, headache, diaphoresis, or palpitations during her hospitalization. It’s unlikely she had a pheochromocytoma, therefore catecholamines and metanephrines were not measured. Additionally, it’s unlikely she had myocarditis as she denied flu-like symptoms, gastrointestinal symptoms, or fever.

Takotsubo cardiomyopathy is typically a transient phenomenon that resolves spontaneously within hours to weeks, requiring only supportive measures to ensure hemodynamic stability. The treatment is directed at managing the specific triggers and risk factors, as well as the management of complications. Due to similarities in initial presentation, patients should be emergently managed with coronary artery disease therapy, including aspirin, intravenous heparin, and beta-blockers. Once coronary artery disease has been ruled out, aspirin should be stopped, but beta-blockers and heparin should be continued [3]. Heparin helps prevent the development of apical thrombus and embolic events and should be continued until regional wall abnormalities resolve. Follow-up for Takotsubo cardiomyopathy should include repeat TTE to assess for the resolution of cardiac wall abnormalities [10]. Beta-blockers help ameliorate the effects of circulating catecholamines on the heart and reduce left ventricular outflow obstruction. Further heart failure therapies should be initiated, including angiotensin-converting enzyme inhibitors/angiotensin II receptor blockers and aldosterone antagonists. Risk factors modification should also be prioritized, including stress reduction and drug cessation as appropriate [3]. Since the patient presented was a fall risk, the hospital team determined she was too high risk for anticoagulation. Additionally, blood pressure-lowering medication, such as beta-blockers and angiotensin-converting enzyme inhibitors, was held as the patient was hypotensive during her hospital stay. She was discharged with plans to be managed outpatient with repeat TTE and monitoring of blood pressure in order to reassess the appropriateness of goal-directed heart failure therapy.

## Conclusions

ALS is a predisposing factor for Takotsubo cardiomyopathy. The patient presented likely developed Takotsubo cardiomyopathy secondary to emotional and physical stress from her terminal ALS diagnosis, and mechanical fall. It’s likely this stress caused catecholamine release leading to cardiomyopathy. This case study highlights a unique case of Takotsubo cardiomyopathy that is linked to ALS and serves as a reminder for clinicians to consider the disease in a patient with ALS who presents with cardiac pathology.
